# Circulating microRNAs 34a, 122, and 192 are linked to obesity-associated inflammation and metabolic disease in pediatric patients

**DOI:** 10.1038/s41366-021-00842-1

**Published:** 2021-05-13

**Authors:** Julia Lischka, Andrea Schanzer, Azadeh Hojreh, Ahmed Ba-Ssalamah, Charlotte de Gier, Isabella Valent, Chike Bellarmine Item, Susanne Greber-Platzer, Maximilian Zeyda

**Affiliations:** 1 Clinical Division of Pediatric Pulmonology, Allergology and Endocrinology, Department of Pediatrics and Adolescent Medicine, Comprehensive Center for Pediatrics, Medical University of Vienna, Vienna, Austria; 2 Department of Biomedical Imaging and Image-guided Therapy, Medical University of Vienna, Vienna, Austria

**Keywords:** Obesity, Metabolic syndrome

## Abstract

**Background:**

Obesity-associated chronic low-grade inflammation leads to dysregulation of central lipid and glucose metabolism pathways leading to metabolic disorders. MicroRNAs (miRNAs) are known to control regulators of metabolic homeostasis. We aimed to assess the relationship of circulating miRNAs with inflammatory modulators and metabolic disorders in pediatric obesity.

**Methods:**

From a pediatric cohort with severe obesity (*n* = 109), clinically thoroughly characterized including diverse routine blood parameters, oral glucose tolerance test, and liver MRI, a panel of 16 circulating miRNAs was quantified using qRT-PCR. Additionally, markers of inflammation TNFα, IL1 receptor antagonist, procalcitonin, CRP, and IL-6 were measured.

**Results:**

Markers of obesity-associated inflammation, TNFα, IL-1Ra, and procalcitonin, all significantly correlated with concentrations of miRNAs 122 and 192. Concentrations of these miRNAs negatively correlated with serum adiponectin and were among those strongly linked to parameters of dyslipidemia and liver function. Moreover, miRNA122 concentrations correlated with HOMA-IR. Several miRNA levels including miRNAs 34a, 93, 122, and 192 were statistically significantly differing between individuals with prediabetes, impaired glucose tolerance, metabolic syndrome, or nonalcoholic fatty liver disease compared to the respective controls. Additionally, miRNA 192 was significantly elevated in metabolically unhealthy obesity.

**Conclusions:**

A miRNA pattern associated with obesity-associated inflammation and comorbidities may be used to distinguish metabolically healthy from unhealthy pediatric patients with obesity. Moreover, these changes in epigenetic regulation could potentially be involved in the etiology of obesity-linked metabolic disease in children and adolescents.

## Introduction

The global obesity epidemic is causing an alarming incidence of metabolic disease already at a young age. Up to 60% of children and adolescents with obesity are estimated to develop metabolic syndrome [1], that is comprised of glucose intolerance, dyslipidemia, hypertension, and abdominal obesity as principal components. Additionally nonalcoholic fatty liver disease (NAFLD) is commonly named as its hepatic manifestation [2]. The clustering of these risk factors are of particular concern in childhood, since their trajectory is already laid out ahead of adulthood [3], leading to increased risk of cardiovascular events and mortality [4]. Therefore, in light of the potentially devastating impact of obesity in children later on in their adult lives, there is urgency in elucidating underlying mechanisms and identifying novel markers for risk stratification and targeted, early treatment.

Chronic low-grade inflammation underlies metabolic dysregulation in obesity. Inflammatory adipokines released from adipose tissue drive chronic low-grade inflammation, which triggers a systemic response potentially leading to metabolic disease [5, 6], while anti-inflammatory and insulin-sensitizing adiponectin is decreased [7]. This is already evident in children, where obesity is associated with chronic low-grade inflammation [7–9] that is maintained into adulthood [9]. Thus, TNFα, IL-6, and CRP are well known markers of obesity-associated inflammation [10–13] and disease [6, 10, 14]. Aberrant levels of CRP and procalcitonin are associated with obesity and adverse metabolic outcomes also in children [2, 8, 15–18]. Circulating IL1 receptor antagonist (IL-1Ra) is a marker of adipocyte inflammatory response [19] described as a strong indicator of an unfavorable metabolic profile [20] and is proposed as marker of obesity-related inflammation in pediatric cohorts [21].

Post-transcriptional control of cytokine production is fine-tuned by microRNAs (miRNAs), small noncoding RNAs that regulate gene expression [22]. miRNAs are also key regulators of metabolic homeostasis, thus aberrant miRNAs expression could contribute to metabolic disease [23–25]. Shifts in miRNA expression have been described in obese phenotypes [26] and a number of miRNAs have been implicated in metabolic disorders including hepatic steatosis or NAFLD [27–30], hypertension [31, 32], insulin resistance [23, 30]. Thus, miRNAs are interrelated to both inflammation and metabolic control. Shifts of inflammatory profile from normal weight to obesity and concomitant disease development have been extensively researched children and adolescents [8, 11, 14], but it has been shown that correlations with metabolic disease in adults did not consistently hold up in pediatric cohorts [9]. This findings highlights the potential differences in pathophysiological disease-triggering processes throughout the life span and thus the need to investigate potential markers and mechanisms in pediatric cohorts [8]. Importantly, the etiology of metabolically healthy vs. metabolically unhealthy obesity (MUO) remains enigmatic to a large extent [33–36]. Epigenetic mechanisms have been suggested to be mechanistically involved in maintenance of metabolic health in obesity [33, 36], but a role of miRNA has not yet been addressed to our knowledge.

Here, we aimed to evaluate the associations between inflammatory and metabolic markers in children and adolescents with severe obesity with a panel of miRNAs that have previously been shown to correlate with markers of metabolic disease mainly in adults or experimental models. We found a network of interrelationships and identified relevant miRNAs as possible crosslinks between metabolic unhealthy phenotype and inflammation in children and adolescents with obesity.

## Methods

### Patients

Patients attending the outpatient clinic for obesity and lipid metabolic disorders at the Department of Pediatrics and Adolescent Medicine at the Medical University of Vienna with a BMI above the 97th percentile (referred to as “severe obesity” [37] throughout this manuscript) were prospectively enrolled in this explorative study. Eligible for this study were all patients between 9 and 19 years old. Patients were excluded if one or more of the following exclusion criteria were met: secondary causes for obesity e.g. endocrine disorders, genetic, syndromic, and drug-induced obesity; treatment with drugs associated with elevated liver enzymes and if other causes for liver disease were present (e.g. Wilson’s disease, hepatitis infection). Of 124 eligible patients, 109 were included in the study. 15 patients were excluded, because of incompliance with study protocol.

All study participants underwent physical examination. Medical history, clinical and laboratory data was collected for all study participants. Anthropometric measures were taken by standardized methods by the same two nurses throughout the study. BMI and the respective percentiles were calculated. Serum and plasma samples were taken in an overnight fasting state and, for nonroutine parameters, frozen at −80 °C until analysis. Homeostasis model of insulin resistance (HOMA-IR) was calculated according to Matthews et al.: fasting insulin (µU/ml) × fasting glucose (mg/dL)/405 [38].

Prediabetes was defined as fasting glucose ≥100 mg/dl. Essential hypertonia was determined with 24-h blood pressure profile. Oral glucose tolerance test (OGTT) was conducted according to the guideline of the German Working Group on Obesity in Childhood and Adolescence and classified as impaired glucose tolerance (IGT) if glucose was between 140 and 199 mg/dl after 2 h [39]. Of note, OGTT results are reported for 81 of 109 children. Metabolic syndrome was defined by the IDF criteria (presence of abdominal obesity (waist circumference (WC) ≥ 90th percentile) and 2 or more of the following criteria: serum triglycerides ≥150 mg/dl, HDL-cholesterol (HDL-C) < 40 mg/dl, systolic blood pressure (SBP) ≥ 130 mmHg or diastolic blood pressure (DBP) of ≥85 mmHg and fasting glucose ≥100 mg/dl or known type 2 diabetes (T2D) mellitus) [40]. Metabolically healthy obesity (MHO) was distinguished from MUO according to the consensus-based definition by Damanhoury et al: HDL-C > 40 mg/dl, triglycerides ≤150 mg/dl, systolic and diastolic blood pressure ≤90th percentile, fasting glucose ≤100 mg/dl [35]. Accumulation of liver fat was quantified by magnetic resonance imaging-proton density fat fraction (MRI-PDFF) and classified as NAFLD if it exceeded 5.1% [41]. Control groups for statistical testing consisted of all children and adolescents without the respective comorbidities or diseases.

### miRNA extraction and quantification

A panel of 16 miRNAs was selected (Supplementary Table 1) due to published association with lipid and glucose metabolism and related disorders [23–25, 30, 32]. Circulating miRNA expression was analyzed after purification of RNA from serum using the miRNeasy Kit (Qiagen, Hilden, Germany). cDNA was synthesized with qScript miRNA cDNA Synthesis Kit (Quantabio, Beverly, MA, USA). miRNA quantification was completed by using PerfeCTa SYBRGreen SuperMix Low ROX (Quantabio) in real-time qRT-PCR. Supplementary to the commercially available kits, oligonucleotides for selected miRNAs (Supplementary Table 1) were purchased from Eurofins. Obtained *C*_T_ values of miRs of interest were multiplied with −1 and normalized to the mean of *C*_T_ of miRNAs 16, 24, 25, and 26 that served as internal controls [42, 43]. Obtained −Δ*C*_T_ values give the logarithmic relative expression.

### Laboratory parameters

TNFα and IL-1Ra were evaluated via ELISA (enzyme-linked immunosorbent assay) according to the manufacturer’s (Quantikine ELISA Kit R&D Systems, Minneapolis, MN, USA).

### Statistics

Data are presented as means ± standard deviations [SD] unless otherwise indicated. Continuous variables were assessed by Pearson correlation and student’s *t*-test or ANOVA if normally distributed. Parameters with skewed distributions were appropriately log-transformed prior to the analyses; if normal distribution was not achieved, respective nonparametric statistics were used.

Correlation tests were calculated adjusted for sex, age, and pubertal stage individually. Collinearity between parameters was controlled with the variation inflation factor (VIF). All VIFs were < 2.1 and thus accepted as low collinearity (VIF < 5) [44]. A two-sided *p* value under 0.05 was considered statistically significant. The confidence interval was set at 95%. Since this study is of explorative character, we did not adjust for multiple testing.

All statistical analyses were performed using IBM SPSS Statistics for Windows, version 25 (IBM Corp., Armonk, N.Y., USA).

## Results

### Characteristics of the study cohort

109 patients were included in the study. The characteristics of the study subjects are shown in Table 1. Mean age was 13.1 ± .2.7 years. About one-fifth (19.3%) had prediabetes and 13.9% had metabolic syndrome. Manifest T2D was present in 4.6% of children and liver steatosis (NAFLD) was the most frequent observed comorbidity in more than half of study patients (57.8%). Altogether, about 75% of the cohort could be regarded as metabolically unhealthy (Table 1).

**Table 1:** Anthropometric and clinical characteristics of study subjects.

	Count (*n* = 109)
Sex (male/female)	37/72
Puberty (prepubertal/pubertal)	31/78
Prediabetes	21 (19.3%)
Impaired glucose tolerance (IGT)	7 (8.6%)
Metabolic Syndrome	15 (13.9%)
Metabolically unhealthy obesity (MUO)	81 (74.1%)
Type 2 diabetes	5 (4.6%)
Hypertonia	16 (15.0%)
NAFLD	63 (57.8%)

	Mean (±SD)
Age	13.1 ± 2.7
BMI *z*-score	2.8 ± 0.5
Waist circumference [cm]	106.5 ± 15.3
Fasting glucose [mg/dl]	84.6 ± 9.7
Insulin [µU/ml]	24.6 (14.9, 33.9)^a^
HOMA-IR	6.3 ± 5.3
MRI-PDFF [%]	8.0 (2.0, 19.5)^a^
AST [U/l]	34.5 ± 18.6
ALT [U/l]	46.6 ± 47.0
GGT [U/l]	24.9 ± 18.7
Triglycerides [mg/dl]	128.2 ± 77.7
Cholesterol [mg/dl]	168.0 ± 31.2
HDL-C [mg/dl]	43.4 ± 10.5
LDL [mg/dl]	99.9 ± 26.3
CK-18 [U/l]	175.7 ± 200.0
CRP [mg/dl]	0.6 ± 0.5
IL-6 [pg/ml]	3.6 ± 2.2
Procalcitonin [ng/ml]	0.04 ± 0.02
TNFα [pg/ml]	1.1 ± 0.4
IL-1Ra [pg/ml]	1,359 ± 2,212

^a^Skewed distribution thus median and interquartile range are presented.

### Associations between circulating miRNA levels and inflammatory parameters

Table 2 summarizes the correlations of miRNAs with inflammatory parameters sorted by the strength of correlation with adiponectin as the central link between adipose-tissue inflammation with systemic inflammation and insulin resistance [5, 7]. Interestingly, markers of obesity-associated inflammation, adiponectin, TNFα, IL-1Ra, and procalcitonin, all significantly correlated (in case of adiponectin negatively) with miRNAs 122 and 192 (Table 2). Moreover, miRNA 34a correlated with TNFα and procalcitonin. When adjusting for age, sex, and pubertal stage, correlations with adiponectin, IL-1Ra and procalcitonin remained significant, while the correlation of miRNA 122 and TNFα did not when adjusting for sex. The traditional markers of inflammation CRP and IL-6 were associated only with miRNA 98 expression and did not correlate with any other miRNA; when adjusting for sex, the correlation with IL-6 loses statistical significance (Table 2).

**Table 2:** Correlations of miRNAs with inflammatory profile.

	Adiponectin [µg/ml]	TNFα [pg/ml]	IL-1Ra [pg/ml]^a^	Procalcitonin [ng/ml]^a^	CRP [mg/dl]^a^	IL-6 [pg/ml]^a^
miRNA 192	−0.31**	0.26*	0.22*	0.39**	−0.15	−0.11
miRNA 122	−0.27**	0.25*^b^	0.24*	0.46**	−0.11	−0.11
miRNA 34a	−0.19	0.22*	0.19	0.27**	−0.11	−0.07
miRNA 193b	−0.17	0.16	0.17	0.20*	0.02	−0.03
miRNA 33b	−0.16	0.16	−0.04	0.22*	−0.05	−0.08
miRNA 27b	−0.14	−0.07	0.10	0.08	−0.06	−0.07
miRNA 23a	−0.06	−0.18	0.03	−0.03	−0.01	−0.02
miRNA 15a	−0.05	−0.08	−0.10	−0.04	−0.08	−0.07
miRNA 1290	−0.05	0.20	0.16	0.35**	−0.01	−0.01
miRNA 33a	−0.03	−0.11	−0.13	0.07	−0.05	−0.09
miRNA 98	0.04	0.01	−0.01	0.07	−0.24*	0.20*^b^
miRNA 144-3p	0.05	−0.04	−0.07	−0.04	0.04	0.02
miRNA 19	0.08	−0.05	−0.21*	0.07	0.03	−0.02
miRNA 93	0.10	−0.14	−0.12	0.04	0.02	0.06
miRNA 197	0.10	−0.06	−0.05	0.01	−0.05	−0.16
miRNA 144-5p	0.14	−0.01	−0.15	−0.03	−0.01	−0.06

^a^Spearman rank correlation coefficients and Pearson correlation coefficients for skewed and normally distributed values, respectively. *p < 0.05; **p < 0.01.

*p* > 0.05 if adjusted for ^b^sex.

Bold values indicate statistical significance.

Hence, our findings indicate that selected miRNAs are linked to obesity-associated inflammatory parameters. Next, we aimed to determine if those inflammation-related miRNAs were also linked to metabolic markers in our cohort.

### miRNA levels relate to metabolic profile

Correlations of miRNAs with metabolic profile are presented in Table 3. miRNAs 33b, 34a, 122, 192, 193b, and 1290 were linked strongly to parameters of dyslipidemia and liver function (Table 3) and only miRNA122 correlated significantly with HOMA-IR. When adjusting for age, sex and pubertal stage, correlations remained intact for all with the exception of the correlation between miRNA 193b and miRNA 93 with CK-18 and miRNA 33a with triglycerides.

**Table 3:** Correlations of miRNAs with metabolic profile.

	HOMA-IR^a^	Triglycerides [mg/dl]^a^	Cholesterol [mg/dl]	MRI PDFF [%]^a^	ALT [U/l]^a^	CK-18 [U/l]^a^
miRNA 192	0.16	0.19*	0.43**	0.38**	0.51**	0.40**
miRNA 122	0.30**	0.24*	0.33**	0.52**	0.65**	0.52**
miRNA 34a	0.16	0.20*	0.24*	0.37**	0.31**	0.33**
miRNA 193b	0.05	0.12	0.21*	0.31**	0.26**	0.26**^b,c,d^
miRNA 33b	0.11	−0.01	0.28**	0.24*	0.25**	0.17
miRNA 27b	−0.03	0.11	0.15	0.1	−0.01	0.11
miRNA 23a	−0.13	−0.02	−0.01	−0.04	−0.06	−0.03
miRNA 15a	−0.08	0.04	−0.02	−0.16	−0.09	−0.15
miRNA 1290	−0.10	0.06	0.10	0.22*	0.25**	0.18
miRNA 33a	−0.09	−0.20*^b,c,d^	0.03	−0.02	0.05	−0.01
miRNA 98	−0.13	−0.01	−0.01	−0.02	−0.01	−0.01
miRNA 144-3p	−0.06	−0.07	−0.1	−0.15	0.02	−0.04
miRNA 19	−0.07	−0.08	0.04	−0.11	0.01	−0.14
miRNA 93	−0.11	−0.16	−0.09	−0.14	0.02	−0.21*^b,c,d^
miRNA 197	−0.01	−0.15	−0.13	0.03	0.03	0.01
miRNA 144-5p	−0.08	−0.01	−0.06	0.03	−0.09	−0.03

^a^Spearman rank correlation coefficients and Pearson correlation coefficients for skewed and normally distributed values, respectively. *p < 0.05; **p < 0.01

*p* > 0.05 if adjusted for ^b^age, ^c^sex, ^d^pubertal stage.

Bold values indicate statistical significance.

**Fig. 1:**
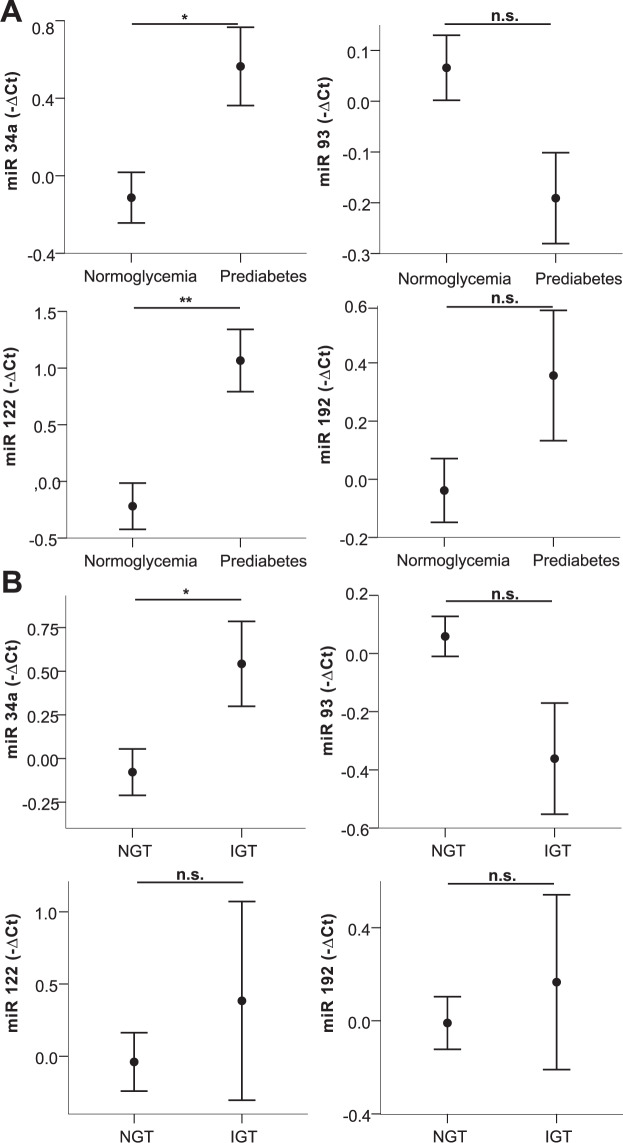
Circulating miRNA profiles in impared glucose metabolism. miRNA 34a, 93, 122, and 192 levels in plasma in children with/without prediabetes (**A**) and with/without impaired glucose tolerance (**B**) are shown. Mean values of indicated miRNA relative expression (−∆Ct values) and error bars indicating SEM are shown. *p < 0.05; **p < 0.01.

**Fig. 2:**
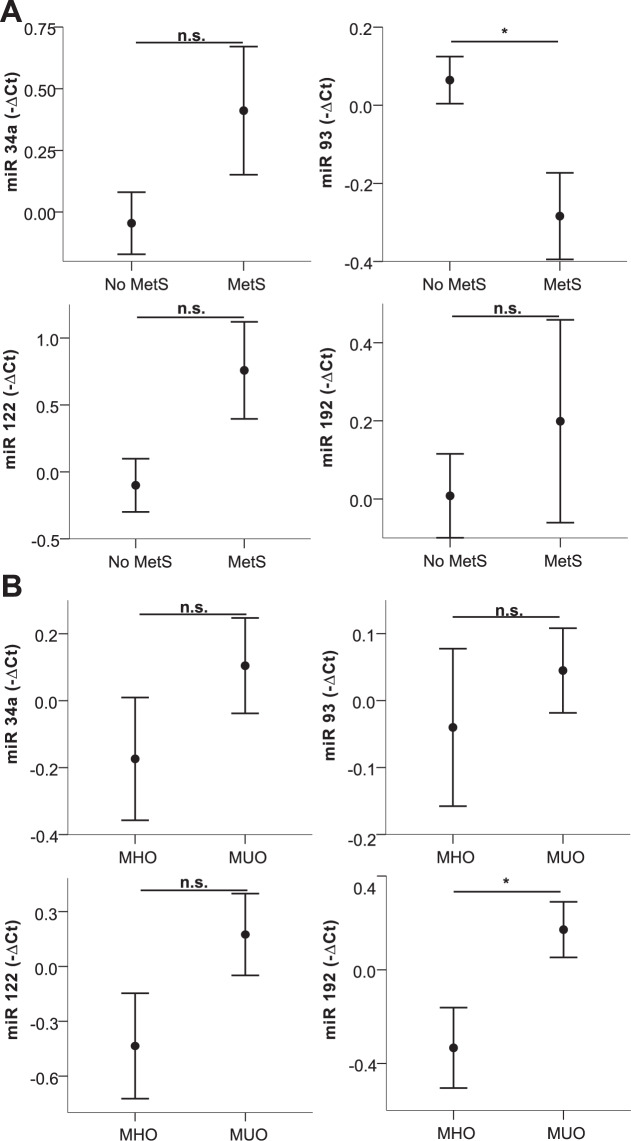
Circulating miRNA profiles in metabolic syndrom und metabolically unhealthy obesity. miRNA 34a, 93, 122, and 192 levels in plasma in children with/without metabolic syndrome (**A**) and MHO/MUO (**B**) are shown. Mean values of indicated miRNA relative expression (−∆Ct values) and error bars indicating SEM are shown. *p < 0.05; **p < 0.01.

**Fig. 3:**
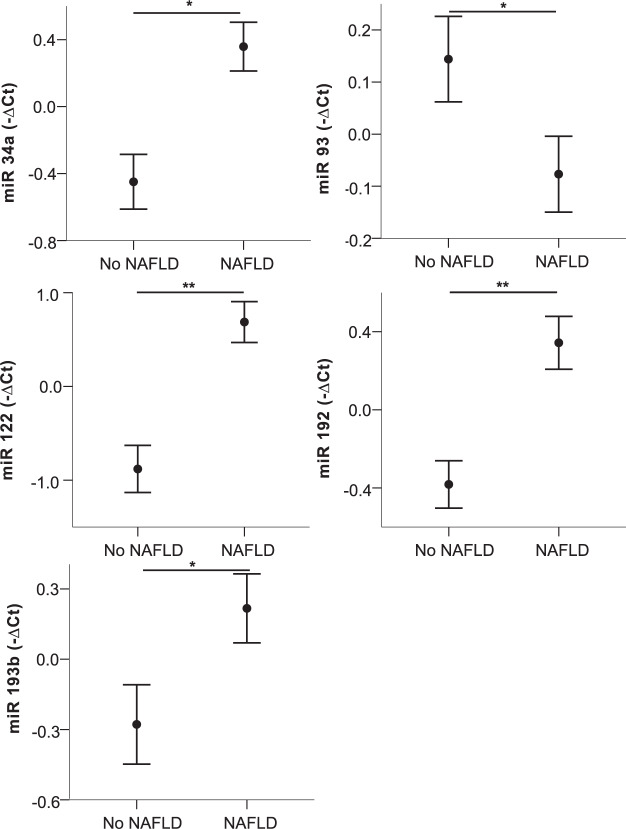
Circulating miRNA profiles in NAFLD. miRNA 34a, 93, 122, 192, and 193b levels in plasma in children with/without NAFLD. Mean values of indicated miRNA relative expression (−∆Ct values) and error bars indicating SEM are shown. *p < 0.05; **p < 0.01.

Next, we exploratively compared patients with prediabetes, IGT, metabolic syndrome, MUO, NAFLD, and hypertension to respective controls. Of note, of the investigated miRNAs, only higher levels of miRNA 192 (*p* = 0.05, Supplementary Table 2) were detected in children with essential hypertension compared to children with normal blood pressure. Circulating levels of miRNAs 34a, 93, 122, and 192 were significantly differing in two or more of these conditions (Supplementary Table 2) and are, therefore, shown in Figs. 1, 2 and 3. Analyzing impaired glucose metabolism, Fig. 1 shows alterations of all miRNAs in prediabetes, and significant higher levels of miRNA 34a in IGT as measured by OGTT, whereas the decrease of miRNA 93 just missed statistical significance (*p* = 0.07). In metabolic syndrome on the other hand, significant alterations of miRNA 93 and for 192 in a metabolically unhealthy obese phenotype were revealed (Fig. 2), additionally, a trend for miRNA 122 (MetS *p* = 0.10; MUO *p* = 0.15) and 34a (MetS *p* = 0.17) is also apparent, although not statistically significant. Of note, higher levels of miRNA 34a, 122, and 192 where associated with the presence of the respective comorbidities, while higher levels of miRNA 93 seem to relate to a healthy phenotype. Figure 3 demonstrates the marked increase in all four miRNAs in NAFLD, and additionally miRNA 193b, that correlated strongly also to liver parameters. Besides these alterations, the significant decrease of miRNA 15a and miRNA 19 in IGT and metabolic syndrome, respectively, were remarkable (Supplementary Table 2). To evaluate a possible impact of puberty on these results, all miRNA expressions were tested for differences between the prepubertal and the pubertal groups, no differences were found. Analyzing the impact of sex, only miRNA 122 was found to differ between males and females. Therefore, the group analyses were performed also for the sexes separately showing similar results with the exception of IGT, which markedly differed between males and females (Supplementary Fig. 1).

## Discussion

Unraveling the role of epigenetic regulation by miRNAs in metabolic health and disease may contribute to the understanding of the dysregulation of inflammatory and metabolic pathways in obesity. The present explorative study revealed miRNAs elevated in metabolic disorders in youth with obesity. Among those, we showed for the first time that miRNA 34a, 122, and 192 were additionally linked to obesity-associated inflammatory markers TNFα, IL-1Ra, adiponectin, and procalcitonin [2, 15, 16, 18],

Circulating miRNAs 122 and 192 have been previously suggested to promote inflammation in the context of liver steatosis [28, 45]. At the molecular level, for miRNA 192 a role of crosslinking inflammation with metabolism may be explained by the previously shown miRNA 192 mediated control of insulin as well as inflammatory signaling pathways in several tissue types [46]. Similarly, miRNA 122 has been shown to be involved in the control of TNFα expression [47]. On another note, cytokines like TNFα and IL-1β were suggested to trigger hepatic secretion of miRNAs 122 and 192 into circulation [28]. Thus, the causal mechanisms behind the observed correlations are still unclear and need to be further investigated.

Addressing another inflammation-associated miRNA, miRNA 34a was previously reported to induce TNFα [48], which is in line with our observation that miRNA 34a correlated with TNFα levels. This underlines its potential role in inflammatory processes such as the situation in obesity. Another interesting marker for inflammatory activity of body fat, procalcitonin [18], correlated strongly with miRNA 34a as well as miRNAs 122 and 192. A possible mechanistic relationship remains to elucidated, but the association shown here supports a role for procalcitonin in obesity-induced inflammation and resulting disorders [18].

Extensive efforts have been made in research studying obesity [1]. Nevertheless, the role of miRNAs remains enigmatic. Initial studies exploring the role of miRNAs in regulation of metabolic pathways reported associations of dyslipidemia with miRNA 33b, 34a, 192, and 193b in adults [24, 49–52], which could be confirmed in our pediatric cohort. Additionally to lipid homeostasis, glycemic control seems to be influenced by miRNAs [53]. Particularly altered levels of miRNA 122, 34a, and 93 were apparent in children with prediabetes in our cohort. Moreover, we here report a novel association between IGT as measured by OGTT and miRNA 34a and 93 expression. The potential central role of miRNA 93, 122, and 192 in metabolic control is further strengthened by our finding that they are altered in metabolic syndrome or MHU. The novel, strong relationship of miRNA 93 to obesity-related metabolic disease in our pediatric cohort is supported by a report linking miRNA 93 to glycemic dysregulation in adults [54].

Since the investigated miRNAs were selected based on their possible involvement in lipid and glucose metabolism [23–25, 30, 32], alterations of their circulating levels in prediabetes, IGT, metabolic syndrome, MUO, and NAFLD in our cohort may have been expected. Nonetheless, for several miRNAs no relationship to metabolic parameters or conditions was detectable: miRNA 23a, 27b, 33a, 33b, 197, 98, and 144 have been previously linked to lipid and/or glucose homeostasis [23–25, 30, 32]. A possible explanation is that—since most previous data were derived from adults—distinct miRNAs might be differentially expressed throughout the lifespan and could play more important roles in disease development later in life. Consequently, it is important to note that an unfavorable miRNA pattern exists already in children with obesity-induced metabolic disorders and includes miRNA 34a, 93, 122, and 192 as crucial markers that may also serve to distinguish healthy from unhealthy phenotypes within pediatric obesity. Importantly in this respect, miRNAs are remarkably stable in blood and, therefore, well suited as biomarkers [55].

Particularly for detection of pediatric NAFLD miRNAs may be interesting biomarkers as previously shown for miRNAs 34a, 122, and 192 [27, 53], with miRNA 122 being a well-known player in liver diseases [27–29, 45]. Here, we also identified miRNA 193b to correlate strongly with liver parameters including CK-18 and liver fat content. Previous studies hinted that miRNA 193b associates with liver disease in rats [56] and is involved in controlling human extracellular matrix genes in liver fibrosis [57]. Moreover, miRNA 193b is linked to adipose tissue inflammation and adiponectin in vitro [58, 59]. Therefore, we hereby provided novel data on a link between circulating miRNA 193b and liver disease in humans that warrants further investigation.

In our analyses we considered sex as a possible confounding factor. We detected sex differences for miRNA 122. The correlation with TNFα was lost when adjusting for sex. A previous study hinted that obese boys might have higher TNFα levels than girls [60]. In our cohort, we could confirm that boys with severe obesity have higher levels of TNFα than girls. Notably, when testing children with comorbidities vs. their respective controls, sex differences were apparent for IGT. Although this could be explained by the small group size in separate analysis, this finding may be very interesting and further investigation of such putatively sex-specific differences is highly desirable in larger cohorts. Same accounts for the impact of puberty, which is not indicated by our data but could emerge by analyzing larger cohorts.

The limitation of the current study is the relatively small sample size. In this respect, children from 9 to 18 years of age were included in this study, spanning a relatively wide range. Although we adjusted for age, sex, and pubertal stage, these might be confounding factors. Since we only included youth with severe obesity from our tertiary care center in Vienna, Austria, the applicability of these results to children with normal weight remains to be determined. Strengths of this study include its prospective character; additionally, strict criteria of inclusion were followed, providing a well-characterized homogenous cohort.

In conclusion, this study describes an unfavorable miRNA profile associated with obesity-related inflammation and metabolic disease in children and adolescents. Particularly miRNAs 34a, 93, 122, and 192 may be attractive biomarkers for the identification of an unfavorable phenotype of pediatric obesity and, hence, could serve for risk stratification followed by targeted early intervention. Moreover, the option of manipulating these miRNAs for treatment purposes may promote further research on the mechanisms underlying the relationship between obesity-related inflammation and metabolic disease.

## Supplementary information

Supplementary Table 1

Supplementary Table 2

Supplemantary Figure 1

## Data Availability

The dataset analyzed during the current study are available from the corresponding author on reasonable request.
