# Supplementary material for: Circulating microRNAs 34a, 122, and 192 are linked to obesity-associated inflammation and metabolic disease in pediatric patients
Source: Int J Obes (Lond). 2021 May 13;45(8):1763–72. doi: 10.1038/s41366-021-00842-1 (PMC8310785; doi:10.1038/s41366-021-00842-1)
Supplement: Supplementary file 3 — Supplemantary Figure 1 [file 41366_2021_842_MOESM3_ESM.pdf]

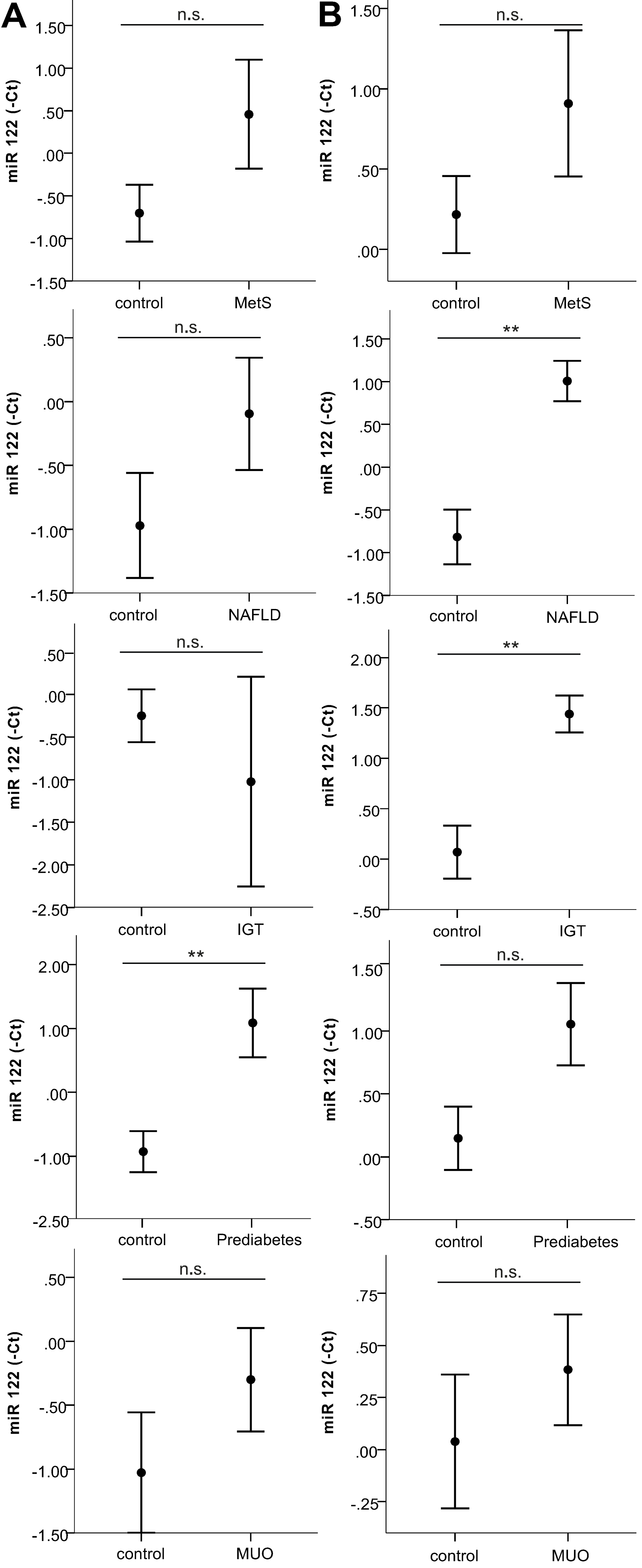

Supplementary Figure 1.
miRNA 122 levels in girls (A) and boys (B) with/without the respective comorbidity. Mean values of miRNA r elative expression (-ΔCt values) and error bars indicating SEM are shown.
*p <0.05; **p <0.01
